# Characterization of sleep quality in Brazilian Jiu-Jitsu fighters

**DOI:** 10.5935/1984-0063.20200001

**Published:** 2021

**Authors:** Francialda Marques Mota Vieira, João Paulo Lima Mouta, Luiz José Frota Solon Júnior, Luiz Vieira da Silva Neto

**Affiliations:** 1 Vale do Acaraú State University, College of Health Science - Sobral - Ceará - Brazil.; 2 Federal University of Ceará, Medicine College of Sobral - Sobral - Ceará - Brazil.

**Keywords:** Sports, Sleep, Performance Tests

## Abstract

**Introduction:**

Sleep quality can positively or negatively inﬂuences the physical performance of Brazilian Jiu-Jitsu ﬁghters; however, the literature does not indicate whether the sleep quality of these ﬁghters is good or poor.

**Objective:**

Characterization of the sleep quality of Brazilian Jiu-Jitsu (BJJ) ﬁghters, stratifying by weight category and color belts.

**Material and Methods:**

A descriptive study that featured with 175 BJJ ﬁghters, the Pittsburgh Sleep Quality Index (PSQI) was applied to assess sleep quality. Mean, standard deviation and 95% conﬁdence interval of mean were used to characterize the data.

**Results:**

All groups, regardless of weight category or color rank, were classiﬁed as poor sleepers (PSQI=5), which indicates a low sleep quality.

**Conclusion:**

The sleep of BJJ ﬁghters is characterized as poor, which may indicate negative outcomes in technical and physical performance.

## INTRODUCTION

Brazilian Jiu-Jitsu (BJJ) is a combat sport that is organized by weight categories and color belts. The aim in its practice is to defeat the opponent through submission, using specific techniques such as joint keys, strangles and immobilizations^1^. For these techniques to be applied there is a need for high levels of strength and power^2^, thus promoting benefits to performance in this sport.

It is known that sports performance can be influenced by both, positive^3^ or negative^4^ sleep quality of the athlete. The literature indicates that in athletes who modify some sleep-related factors, such as sleep duration, temperature and room brightness^5^, they promote better sleep quality and, consequently, better sports performance.

However, poor sleep leads to less sleep quality^4^, thus affecting aspects such as strength, power, attention and concentration^6^, which in turn are determining factors for a good performance in sports, specifically in the case of BJJ, that depends on these aspects for your success^7^.

Therefore, it is important to characterize the sleep quality of BJJ fighters, since this information allows planning strategies to improve performance, recovery, and even to monitor the physical health of these fighters. This would assist teams involved in training and follow up of these fighters.

The aim of this study is to characterize the sleep quality of BJJ fighters, stratifying by weight category and color belts.

## MATERIAL AND METHODS

### Participants

The total number of participants in this study was 175 BJJ fighters of all color belts. The mean age was 30.6±7.5 years, mean body mass 84.8±14.7kg, the mean trainings per week 4.5±2.6 times, and training duration 88.8±27.4 min. All participants were training continuously for at least 6 months.

All fighters were invited to their training center. After receiving an initial explanation, and giving their informed consent through the free and informed consent form, the fighters responded to the instrument privately, 30 minutes before the training and without interruption. All fighters were without exercise for 48 hours before answering the questionnaire.

This research was approved by the Human Research Ethics Committee of Vale do Acaraú State University with the number 3.095.508.

### Pittsburgh Sleep Quality Index

To assess the subjective sleep quality Pittsburgh Sleep Quality Index (PSQI) developed by Buysse et al.^8^ and validated for Brazil later by Bertolazi et al.^9^ was used. PSQI consists of 19 items, which are divided into seven subscales: sleep quality, sleep latency, sleep duration, habitual sleep efficiency, sleep disturbances, use of sleep medication, and daytime dysfunction, each subscale can range from 0 to 3 scores. PSQI total scores can range from 0 to 21 scores. Values below 5 scores indicate good sleep quality, while values equal to or greater than 5 scores indicate poor sleep quality, so that it should be considered that the higher the PSQI score, the worse the sleep quality.

### Statistical analysis

As this is a characterization research, a descriptive statistics technique was used. We obtained mean and standard deviation to data characterization. Confidence intervals were constructed from the 95% for mean. All data were analyzed in the statistical package for social sciences (SPSS 23.v).

## RESULTS

By stratifying BJJ fighters by weight category, we have light feather (N=6), feather (N=30), light (N=21), middle (N=27), medium heavy (N=27), heavy (N=22), super heavy (N=23) and ultra heavy (N=19). Most fighters were from the feather category that is up to 70kg.

The fighters were categorized by the following color belts: white (N=26), blue (N=56), purple (N=34), brown (N=20) and black (N=39). Most fighters are in the blue belt.

All BJJ fighters scored above 5 scores, which characterizes them as poor sleepers (Table 1). It is noteworthy that only in the super heavy weight category the CI, was outside the range of 5 scores.

**Table 1:** Characterization of PSQI by the Weight category.

	PSQI	
	M(SD)	95% CI for M
Light Feather	7.1 ±1.9	5.1 - 9.2
Feather	7.±2.5	6 - 7.9
Light	7.2 ±2.8	5.9 - 8.5
Middle	8.1 ±2.5	7.1 - 9.1
Medium Heavy	6.4 ±2.3	5.4 - 7.3
Heavy	7.2 ±3.5	5.6 - 8.7
Super Heavy	5.9 ±2.7	4.7 - 7.1
Ultra Heavy	7.9 ±3.4	6.2 - 9.5
**Total**	**7.1 ±2.8**	**6.7 - 7.5**

Legend: M (Mean), SD (Standard Desviation), CI (Confidence Interval)

When the characterization of sleep was done through the color belts, all fighters are classified as poor sleepers (Table 2).

**Table 2:** Characterization of PSQI by Color Belt

	PSQI	
	M(SD)	95% CI for M
Light Feather	7.1 ±1.9	5.1 - 9.2
Feather	7.±2.5	6 - 7.9
Light	7.2 ±2.8	5.9 - 8.5
Middle	8.1 ±2.5	7.1 - 9.1
Medium Heavy	6.4 ±2.3	5.4 - 7.3
Heavy	7.2 ±3.5	5.6 - 8.7
Super Heavy	5.9 ±2.7	4.7 - 7.1
Ultra Heavy	7.9 ±3.4	6.2 - 9.5
**Total**	**7.1 ±2.8**	**6.7 - 7.5**

Legend: M (Mean), SD (Stadart Desviation), CI (Confidence Interval)

## DISCUSSION

The study of Vargas et al.^10^ shows that a relationship of poor sleep quality, having a direct influence on body weight gain, which has a negative impact on this aspect of health, and what would explain the poor sleep quality of fighters with higher body weight. However, our results show that regardless of weight category, all fighters have poor sleep quality, including the lightest ones (light feather). Therefore, the literature brings multifactorial processes, such as mood states changes, bad thoughts and use of electronic devices as promoters of a poor sleep quality^11^. These processes justify the results found in all weight categories.

BJJ color belts are subjective indicators of technical level^12^. So, the data in Table 2 shows that regardless of whether they have the first (white belt) or the last (black belt), all fighters are classified as poor sleepers. This outcome is corroborated by the literature, because athletes of different modalities and regardless of the technical level have been presenting a poor sleep quality^13^.

The poor sleep quality in BJJ fighters analyzed in this research may cause loss to their performance, such as the reduction of strength, power, attention and concentration^6^, which negatively impact on aspects related to technical learning and task execution.

Another factor that must be considered is that poor sleep quality is directly linked to the higher risk and incidence of injuries^14^, thus aggravating the high incidence of injuries in BJJ fighters in both, training and competitions^15^.

Considering the relevance of our findings, we cannot disregard the main limitation of this study, which is due to the use of only a subjective and indirect instrument (PSQI) to measure sleep quality, which makes necessary further analysis of other aspects such as phase duration, exact time to sleep and total time in bed. However, it should be noted that the sample used in this study is relevant, and may be one of the largest samples of BJJ fighters in the world. Besides that, there is no literature so far. Thus, data such as those obtained in this study can help multidisciplinary teams in monitoring the performance of these fighters.

## CONCLUSION

BJJ fighters are poor sleepers regardless of weight category or color belts. From this point, there is information that can support the professionals (technicians, psychologists, physiotherapists and medicine doctors) involved with these fighters, to promote better sleep quality and consequently a better performance.

## References

[1] Andreato LV, Lara FJD, Andrade A, Branco BHM (2017). Physical and physiological profiles of Brazilian Jiu-Jitsu athletes: a systematic review. Sports Med Open.

[2] Spano M, Risucci DA, Etienne M, Petersen KH (2019). Epidemiology of sports related concussion in Brazilian Jiu-Jitsu: a cross-sectional stud. Sports (Basel).

[3] Nédeléc M, Halson S, Delecroix B, Abaidia AE, Ahmaidi S, Dupont G (2015). Sleep hygiene and recovery strategies in elite soccer players. Sports Med.

[4] Biggins M, Cahalan R, Comysns T, Purtill H, O'Sullivan K (2018). Poor sleep is related to lower general health, increased stress and increased confusion in elite Gaelic athletes. Phys Sportsmed.

[5] Knufinke M, Nieuwenhuys A, Geurts SAE, Coenen AML, Kompier MAJ (2018). Self-reported sleep quantity, quality and sleep hygiene in elite athletes. J Sleep Res.

[6] Simpson NS, Gibbs EL, Matheson GO (2017). Optimizing sleep to maximize performance: implications and recommendations for elite athletes. Scand J Med Sci Sports.

[7] Andreato LV, Santos JF, Esteves JV, Panissa VL, Julio UF, Franchini E (2016). Physiological nutritional and performance profiles of Brazilian Jiu-Jitsu athletes. J Hum Kinet.

[8] Buysse DJ, Reynolds CF, Monk TH, Berman SR, Kupfer DJ (1989). The Pittsburgh Sleep Quality Index: a new instrument for psychiatric practice and research. Psychiatry Res.

[9] Bertolazi NA, Fagondes SC, Hoff LS, Dartora EG, Miozzo ICS, Barba MEF (2011). Validation of Brazilian Portuguese version of the Pittsburgh Sleep Quality Index. Sleep Med.

[10] Vargas PA, Flores M, Robles E (2014). Sleep quality and body mass index in college students: the role of sleep disturbances. J Am Coll Health.

[11] Hoshikawa M, Uchida S, Hirano Y (2018). A subjective assessment of the prevalence and factors associated with poor sleep quality amongst elite Japanese athletes. Sports Med Open.

[12] Øvretveit K (2018). Anthropometric and physiological characteristics of Brazilian Jiu-Jitsu athletes. J Strength Cond Res.

[13] Swinbourne R, Gill N, Vaile J, Smart D (2016). Prevalence of poor sleep quality, sleepiness and obstructive sleep apnoea risk factors in athletes. Eur J Sport Sci.

[14] Watson AM (2017). Sleep and athletic performance. Curr Sports Med Rep..

[15] Petrisor BA, Del Fabbro G, Madden K, Khan M, Joslin J, Bhandari M (2019). Brazilian Jiu-Jitsu injury in training survey. Sports Health.

